# Study on the Characteristics of Gas Molecular Mean Free Path in Nanopores by Molecular Dynamics Simulations

**DOI:** 10.3390/ijms150712714

**Published:** 2014-07-18

**Authors:** Qixin Liu, Zhiyong Cai

**Affiliations:** 1 School of Civil Engineering and Architecture, Chongqing University of Science and Technology, Chongqing 401331, China; 2 School of Safety Engineering, Chongqing University of Science and Technology, Chongqing 401331, China; E-Mail: czy677656@gmail.com

**Keywords:** nanopore, gas molecular mean free path, Knudsen number, molecular dynamics simulation

## Abstract

This paper presents studies on the characteristics of gas molecular mean free path in nanopores by molecular dynamics simulation. Our study results indicate that the mean free path of all molecules in nanopores depend on both the radius of the nanopore and the gas-solid interaction strength. Besides mean free path of all molecules in the nanopore, this paper highlights the gas molecular mean free path at different positions of the nanopore and the anisotropy of the gas molecular mean free path at nanopores. The molecular mean free path varies with the molecule’s distance from the center of the nanopore. The least value of the mean free path occurs at the wall surface of the nanopore. The present paper found that the gas molecular mean free path is anisotropic when gas is confined in nanopores. The radial gas molecular mean free path is much smaller than the mean free path including all molecular collisions occuring in three directions. Our study results also indicate that when gas is confined in nanopores the gas molecule number density does not affect the gas molecular mean free path in the same way as it does for the gas in unbounded space. These study results may bring new insights into understanding the gas flow’s characteristic at nanoscale.

## 1. Introduction

For nanoscale gas flows, the flow flux is larger than the value based on classical fluid mechanics theory. Previous investigations [1,2] found that water or gas flows much faster inside a carbon nanotube than in a classical macroscale tube. Due to increased interest in nanofluidics and nanoelectromechanical devices [3], these unusual flow phenomena have attracted much interest from researchers.

When the system size becomes comparable to the mean free path of the molecules, the continuum assumption should break down and the classical Navier-Stokes (N-S) equations with no-slip boundary conditions cannot be directly applied [4]. The Knudsen number is defined as Kn = *λ / H*, where *λ* is the molecular mean free path and *H* is the representative length of the domain. The Knudsen number is widely used to divide the gas flow into four regimes: Kn < 0.001 is the continuum regime, 0.001 < Kn < 0.1 is the slip regime in which the Navier-Stokes equations with the slip boundary condition are appropriate, 0.1 < Kn < 10 is the transition regime and 10 < Kn is the free molecular regime. According to the definition of Knudsen number, the gas’s molecular free path is very important in judging the flow regime. However, the gas molecular mean free path at macroscale and not its effective mean free path is usually used to calculate the Kn number. Thus, it is important to study the effective molecular free path of gas at the nanoscale.

The mean free path concept is also central to most models of the transport phenomenon in gases, such as the momentum, energy and mass exchanges which are essential to the gas flow and heat transfer character at nanoscale space. The most important difference between gas flow at nanopore and macroscale tubes is that the boundary of the nanopore limits the molecular motions in the radial direction. Another important characteristic of gas flow at nanoscale is the uneven gas molecule number density distribution in the whole flow space [5,6]. Simple kinetic theory indicates that the gas molecule’s number distribution would affect the gas molecular mean free path, hence its effective mean free path should be modified due to the limitation of motions in the radial direction and the uneven distribution of gas molecule’s number density. The effective gas molecular mean free path at the nanopore is therefore essential to clarify.

Stops [7] theoretically studied the mean free path of gas molecules bounded by two parallel planes. His study is based on that the possibility of a molecule having free path *r* is described as:

*ψ*(*r*) = *λ*^−1^ exp(*−r / λ*) (1)

The study shows that the mean free path of gas is reduced. Dongari and his co-workers [8] studied the gas mean free path in rarefied gas and their results indicate that molecules perform Levy-type flights under rarefied conditions and the free path of gas molecules follows a power-law distribution. Taking into account the solid boundary effects their further work [9] derived an effective mean free path model for flows confined by planar surfaces, which obtained good agreement with the molecular dynamics (MD) data up to the early transition regime. The mean free path of gas between the cylinders was studied by taking into account the boundary-limit effects on the molecular mean free path for surfaces with both convex and concave curvatures, though some collisions were neglected [10]. The studies above demonstrate that limited studies have been devoted to the gas mean free path at nanoscale. At nanoscale, the gas-solid interaction influences the molecule’s motion, which cannot be neglected. But to our knowledge, there are rare reports on the influences of different gas-solid interaction strengths on the gas molecular mean free path. On the other hand due to the limitations on the gas molecular motion by the nanopore’s surface, the gas molecular free path might be anisotropic. However, gas molecular momentum and energy exchange characteristics are highly dependent on the radial gas molecular mean free path in nanopores, so it is important to study the radial gas molecular mean free path. But to our knowledge, there is a lack of the studies on the radial gas molecular mean free path in nanopores.

Experimental measurements are optimal for these studies; it is however, very difficult to carry out direct measurements on the gas molecular mean free path when it is confined in nanopores. Thus, theoretical analysis based on the kinetic theory is usually carried out to obtain the mean free path. Some assumptions should be made to calculate the gas free path in nanopores by kinetic theory because the interactions between gas molecules and the solid wall must be carefully considered. Besides theoretical calculation, simulation methods such as MD simulation allow us to assess some fundamental molecular properties such as the inter-molecular collisions and the distances between two collisions. MD is one very useful method to get the molecular free path at nanoscale space. This paper presents our numerical experiments on simple gas confined at nanopores, which are the most common and most easily processed. We highlight the profile of gas mean free path and radial mean free path and their relationships with the radius of the nanopores and the gas-solid interaction strength.

## 2. Results and Discussion

In this paper, we present our study results on nanopores of three different radii, which are 16.06, 21.4 and 32.0 nm. For each nanopore, three different gas-solid interaction strengths, that is *c* equals 0.2, 0.4 and 0.6, were applied to study its effects on the gas molecular mean free path.

### 2.1. Mean Free Path of All Molecules in Nanopore

According to the gas kinetic theory, the mean free path can be expressed as Equation (2) for gas at macroscale.

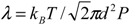
(2)
where *k_B_* is the Boltzmann constant, *d* is the diameter of gas molecule and *P* is the gas pressure. Substituting the corresponding value in our studies we get that the mean free path of argon at the same temperature and density as our simulation cases but at macroscale space is 35.85 nm.

Through calculating the integration described as Equation (3), in which *ψ*(*r*) is the possibility a molecule has a free path between *r* and *r + dr*, we get the mean free path of all molecules in the nanopore, which is represented by *λ_all_* in the following section.

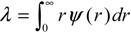
(3)

Table 1 lists the ratio of *λ_all_* to the kinetic theoretical value of the gas mean free path which is represented by *λ*_0_. From Table 1, it can be clearly seen that when gas is confined at the nanopore *λ_all_* is less than its counterpart value at macroscale space. Table 2 gives the comparison between the Knudsen number based on the kinetic theoretic value of mean free path (Kn_0_) and the actual Knudsen number which is based on the effective mean free path. From Table 2 we can see that if the gas mean free path at macroscale space is adopted to calculate the Knudsen number we will get the overestimated Knudsen number which would bring adverse impact on the understanding of flow characteristics at nanoscale.

**Table 1:** Ratio of our simulations results *λ_all_* to the kinetic theoretic value of the gas molecular mean free path.

*λ_all_*/*λ*_0_	*R* = 16.06 nm	*R* = 21.4 nm	*R* = 32.0 nm
*c* = 0.2	0.321	0.388	0.519
*c* = 0.4	0.209	0.248	0.371
*c* = 0.6	0.128	0.179	0.237

**Table 2:** Comparison between the Kn_0_ which is based on the kinetic theoretic value of the gas mean free path and the actual Knudsen number.

Actual Kn	*R* = 16.06 nm	*R* = 21.4 nm	*R* = 32.0 nm
	Kn_0_ = 1.116	Kn_0_ = 0.837	Kn_0_ = 0.560
*c* = 0.2	0.358	0.325	0.291
*c* =0.4	0.233	0.208	0.208
*c* = 0.6	0.143	0.150	0.133

Another conclusion drawn from Table 1 is that both the nanopore’s radius and the gas-solid interaction strength will affect *λ_all_*. According to Table 1, a smaller nanopore and stronger gas-solid interactions will cause shorter *λ_all_*. A smaller nanopore has a larger ratio of surface area to its volume and the wall surface will take more constraints on the gas molecule’s movements so the mean free path naturally becomes smaller. When the gas-solid interaction becomes stronger more gas molecules will adhere to the wall of the nanopore; those molecules will experience shorter travel distance between two successive collisions and hence decrease the mean free path of all molecules in the nanopore.

### 2.2. Profile of Gas Molecular Mean Free Path

In this section, we present our study results on the profile of the gas molecular mean free path along the radius of the nanopore. To get the profile, we divide the nanopore into many concentric annuluses and the width of each annulus depends on its distance from the wall surface of nanopore. For annuluses which are only 2*δ* apart from the wall surface, their width is 0.125*δ* and others have the width of 4*δ*. The purpose of taking different widths is to obtain detailed information about the zone only 2*δ* apart from the wall surface. To get the gas molecular mean free path in each annulus the calculation of Equation (3) is done over each annulus. In this section we present the profile of mean free path including gas molecule’s collisions in all directions, which is expressed as *λ_all−direction_*.

**Figure 1:**
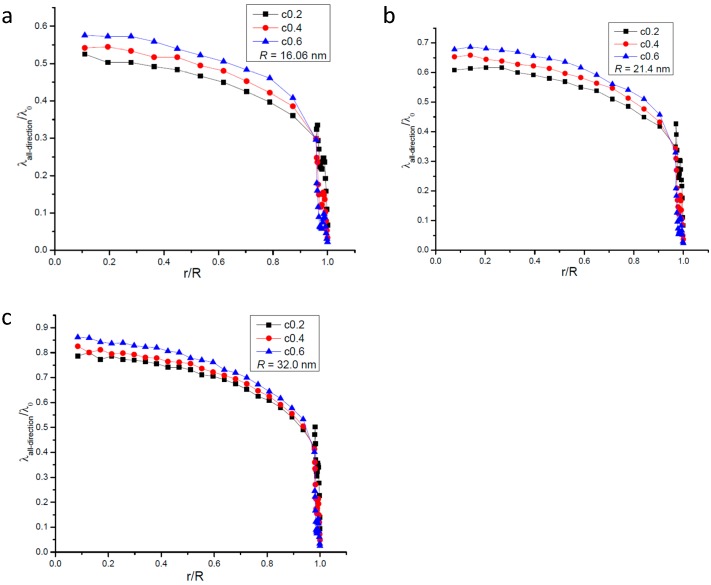
Variation of gas molecular mean free path with the distance from the center of the nanopore. (**a**) *R* = 16.06 nm; (**b**) *R* = 21.4 nm; and (**c**) *R* =32.0 nm.

Our simulation results are shown in Figure 1 in which the longitudinal coordinate represents the ratio of *λ_all−direction_* to the unconfined mean free path *λ*_0_ whose value is 35.85 nm. Figure 1 describes several properties concerning the variation of *λ_all−direction_* with its distance from the center of the nanopore. First, the largest value of the mean free path occurs at the center of the nanopore and it is about the radius of the nanopore. This phenomenon is physically reasonable because the molecules at the center of nanopore have the largest space to move; Second, the mean free path decreases along the nanopore’s radius direction but with different decreasing rates in different regions. For molecules in the region from the center of the nanopore to 2 nm apart from the wall surface, *λ_all−direction_* only declines about 5 nm. However, for molecules which are near the wall surface, their mean free path sharply decreases; Thirdly, like the mean free path of all gas molecules in the nanopore, the gas-solid interaction strength also affects the profile of *λ_all−direction_*. When the gas-solid interaction strength becomes stronger *λ_all−direction_* in the region that suffers no direct effects from the wall surface, becomes larger. This so-called region which suffers no direct effects from the wall surface in this paper means the zone in which gas molecules only experience gas-gas collisions and no collisions between molecules and the wall surface occurs. Stronger gas-solid interactions would cause more gas-wall collisions and hence decrease gas-gas collisions, which make *λ_all−direction_* in the region which suffers no direct effects from the wall surface become larger. This phenomenon can be further illustrated by the molecular collision times data recorded in our simulations. Table 3 gives the comparison of molecule collision times in the region that suffers no direct effects from the wall surface when different gas-solid interaction strengths are applied. It should be noted that the molecule collision times in Table 3 means the average number of gas-gas collisions per unit time. Our MD data shows that when *c* is 0.2 the molecules in the region that suffers no direct effects from the wall surface experience the most gas-gas collisions, so we set it as the comparison standard. For the nanopores with the same radius the value in Table 3 is the ratio of its actual molecule collision times to the value of simulation results when *c* is 0.2. Table 3 clearly illustrates that when the gas-wall interaction strength increases the gas molecules in the region which suffers no direct effects from the wall experience less collisions and hence causes the gas mean free path in this region to increase.

**Table 3:** Comparison of molecule collision times in the zone which suffers no direct effects from the wall between different gas-solid interaction strength.

Comparison of Molecule Collision Times	*R* = 16.06 nm	*R* = 21.4 nm	*R* = 32.0 nm
*c* = 0.2	1	1	1
*c* = 0.4	0.937	0.968	0.981
*c* = 0.6	0.893	0.911	0.947

**Figure 2:**
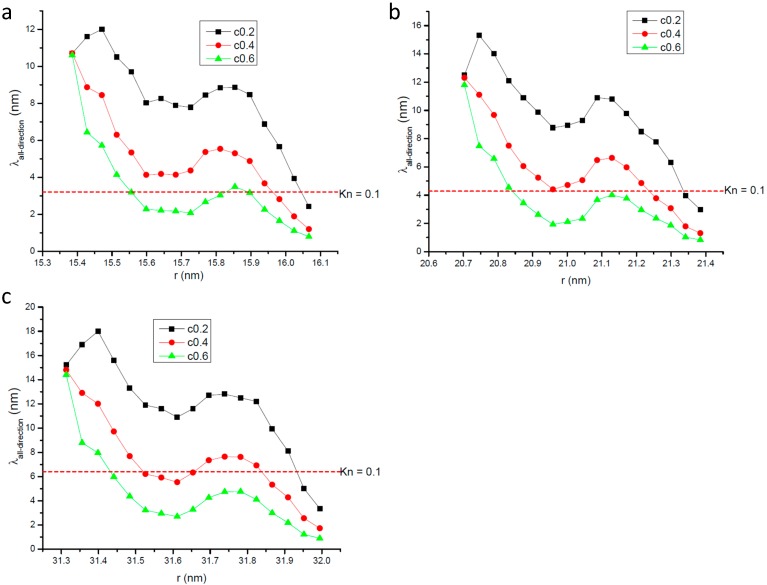
Profile of gas molecular mean free path in the zone 0.7 nm apart from the wall surface of the nanopore; the dotted line means the dividing line of slip regime. (**a**) *R* = 16.06 nm; (**b**) *R* = 21.4 nm; and (**c**) *R* = 32.0 nm.

To further study the variation of mean free path near the wall surface of the nanopore, Figure 2 gives the profile of *λ_all−direction_* within the zone of 0.7 nm apart from the wall surface. Figure 2 shows that in the near-wall zone the mean free path does not vary monotonously and fluctuations can be clearly seen. The least mean free path occurs at the wall surface whose value ranges from about 0.9 to 4.0 nm. When the gas-solid interaction strength is identical, the least value of *λ_all−direction_* for different nanopores is nearly the same, which shows the gas-solid interaction strength plays a key role in determining *λ_all−direction_* at the wall surface.

According to its definition, the value of the Knudsen number should have the same variation tendency with the mean free path. Substituting the mean free path to the definition of Kn, the line of Kn = 0.1 is shown in Figure 2. According to Figure 2 it is interesting that the Knudsen number in the zone near the wall surface of the nanopore is less than 0.1 and in other parts of the nanopore the Knudsen number is greater than 0.1.

### 2.3. Profile of the Radial Gas Molecular Mean Free Path

In macroscale tubes, the gas molecular mean free path is isotropic due to a lack of surface effects on the molecule’s motion. But for a nanopore, the gas molecule’s motion is limited in the radial direction, so is the gas molecular mean free path still isotropic? In this section we will give our simulation results on the radial gas molecular mean free path in nanopores. The radial gas molecular mean free path expressed as *λ_r_* in this paper is defined as the mean distance in the radial direction between gas molecule’s two successive collisions, which includes both gas molecule-gas molecule and gas molecule-wall surface collisions.

**Figure 3:**
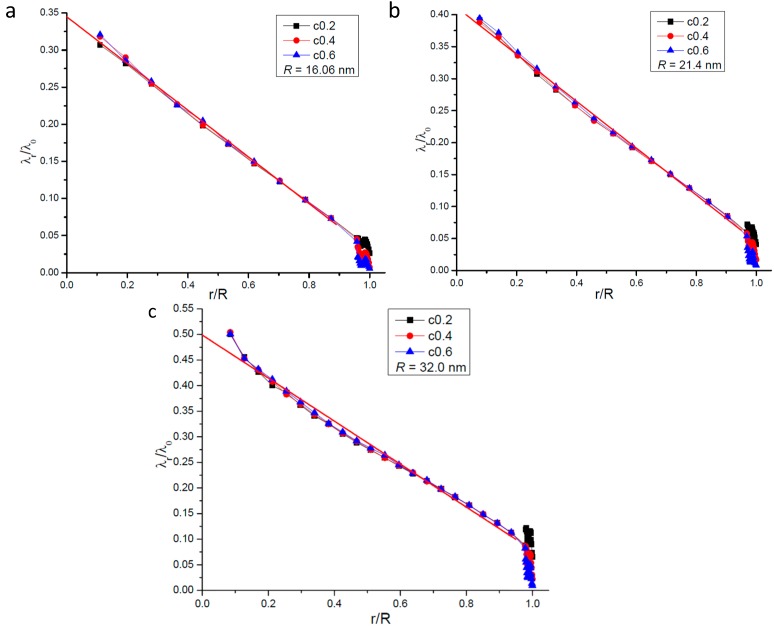
Variation of radial gas molecular mean free path with the distance from the center of the nanopore;the line represents the fitting results. (**a**) *R* = 16.06 nm; (**b**) *R* = 21.4 nm; and (**c**) *R* = 32.0 nm.

Figure 3 gives the variation of radial gas molecular mean free path with the distance from the center of the nanopore. In Figure 3 the longitudinal coordinate stands for the ratio of *λ_r_* to the unconfined mean free path *λ*_0_ whose value is 35.85 nm. In the region far from the wall surface *λ_r_*, like *λ_all−direction_*, also increases with the distance from the wall surface, but it changes linearly with its distance from the center of the nanopore. This change tendency along the radius is different from that of *λ_all−direction_*. The linear fit of relationship between *λ_r_* / *λ*_0_ and *r* / *R* in the region far away from the wall surface is also presented in Figure 3 and it is found that it can be described by one linear function with the form of *λ_r_ / λ*_0_ = *A + B* × (*r / R*). The values of *A* and *B* only vary with the nanopore’s radius. For the same nanopore, even the gas-solid interaction strength is different from the relationship between *λ_r_* / *λ*_0_ and *r* / *R* can be described by the same function.

Comparing Figure 3 with Figure 1, there is great difference seen between the value of *λ_r_* and *λ_all−direction_*. This phenomenon clearly illustrates that the gas molecules’ motions are anisotropic when they are confined in nanopores. The most important point is that the mean free path will affect the momentum and energy exchange, so it could be deduced that the momentum and energy exchange characteristics are also anisotropic in nanopores. The ratio of *λ_r_* to *λ_all−direction_* increases when the nanopore becomes larger but is not been affected by the gas-wall interaction strength. So it seems that the anisotropy of molecular transportation is only affected by the size of the nanopore.

**Figure 4:**
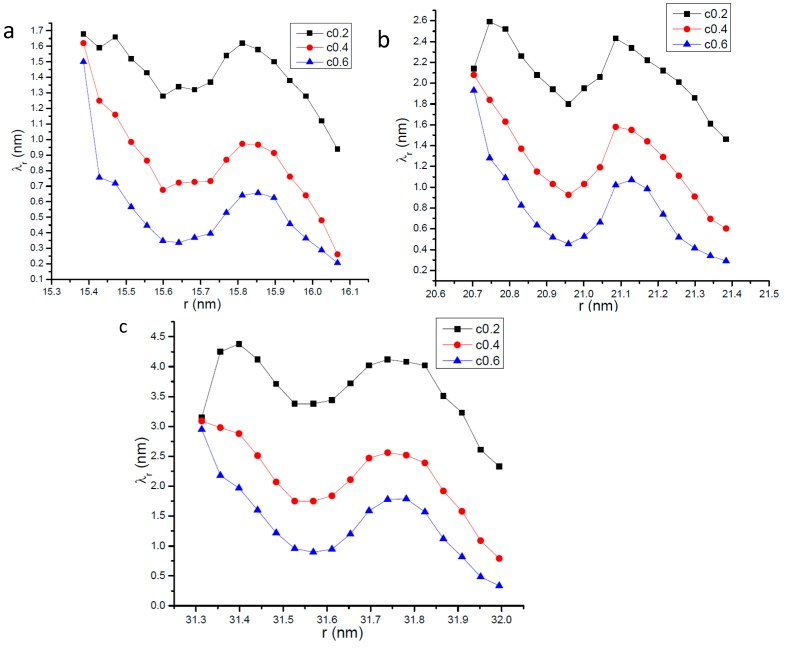
Profile of radial gas molecular mean free path in the zone 0.7 nm apart from the nanopore’s wall surface. (**a**) *R* = 16.06 nm; (**b**) *R* = 21.4 nm; and (**c**) *R* = 32.0 nm.

Figure 4 gives the details of radial gas molecular mean free path in the zone 0.7 nm apart from the nanopore’s wall surface. It shows that the value of *λ_r_* is much smaller than that of *λ_all−direction_* in the same zone. Comparing Figure 4 with Figure 2 we can find that in this zone both *λ_r_* and *λ_all−direction_* show nearly the same change tendency with the distance from the wall surface. This phenomenon illustrates that in the zone directly affected by the wall surface the wall surface’s effects dominate both *λ_r_* and *λ_all−direction_*.

### 2.4. Relationship between Gas Number Density and Molecular Mean Free Path in Nanopores

**Figure 5:**
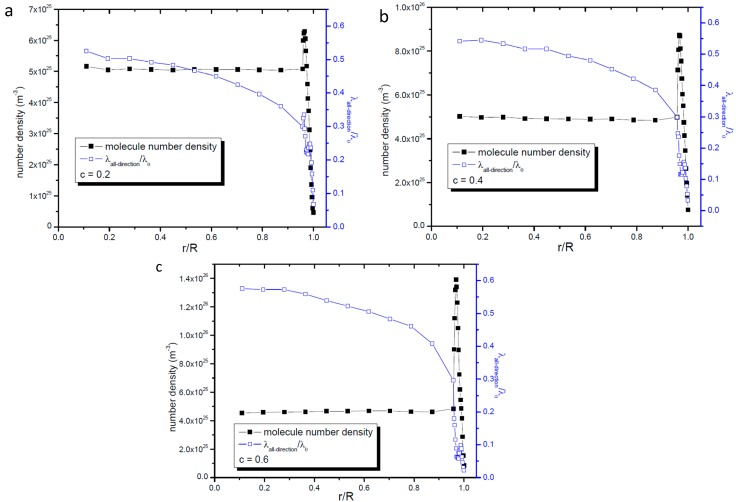
Profiles of gas molecular mean free path and gas number density. (**a**) *c* = 0.2; (**b**) *c* = 0.4; and (**c**) *c* = 0.6.

It is well known that the gas number density shows an uneven distribution near the wall surface when it is confined in nanopores. The above results show that the gas molecular mean free path also varies with its distance from the center of the nanopore, so it is necessary to study the relationship between the gas number density and the gas molecular mean free path. In this paper we take the nanopore whose radius is 16.06 nm as example to study the relationship between the gas number density and the molecular mean free path. Figure 5 shows the profiles of gas number density and molecular mean free path including all collision directions. Figure 5 shows that there is a peak number density value near the wall surface. Unlike the gas molecular mean free path, the gas number density in most spaces of the nanopore does not vary obviously, which illustrates that in the constant density zone the variation of the gas molecular mean free path is not caused by the gas number density.

**Figure 6:**
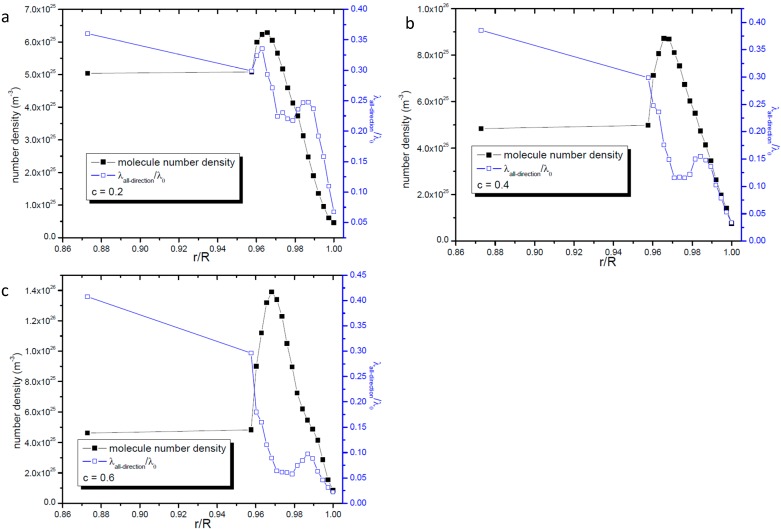
Profiles of gas molecular mean free path and gas number density in the zone 0.7 nm apart from the nanopore’s wall surface. (**a**) *c* = 0.2; (**b**) *c* = 0.4; and (**c**) *c* = 0.6.

To carefully study both the variations of gas number density and molecular mean free path in the zone near the wall surface we give the details in the zone 0.7 nm apart from the nanopore’s wall surface in Figure 6. According to the kinetic theory, for gas molecules in the unbounded space the larger gas number density will reduce the gas molecular mean free path. However, according to Figure 6 at the position where the highest value of gas number density occurs, the gas molecular mean free path dose not show the lowest value. When the number density declines, the gas molecular mean free path also declines. These phenomena illustrate that for gas confined in nanopores the wall surface has stronger effects on the gas molecular mean free path than gas number density does.

**Figure 7:**
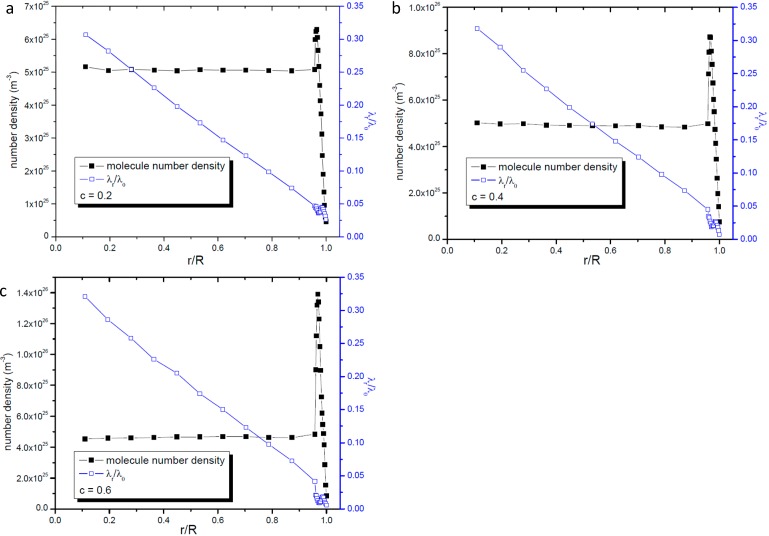
Profiles of radial gas molecular mean free path and gas number density. (**a**) *c* = 0.2; (**b**) *c* = 0.4; and (**c**) *c* = 0.6.

**Figure 8:**
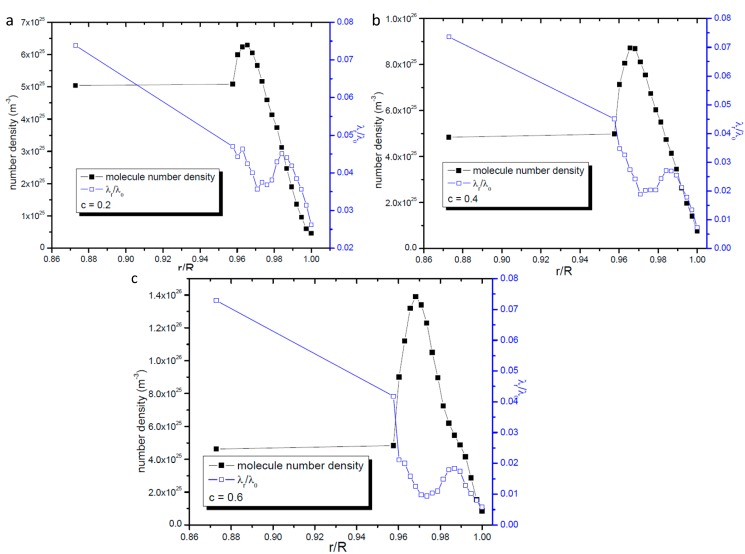
Profiles of radial gas molecular mean free path and gas number density in the zone 0.7 nm apart from the nanopore’s wall surface. (**a**) *c* = 0.2; (**b**) *c* = 0.4; and (**c**) *c* = 0.6.

As far as the radial gas molecular mean free path is concerned, Figure 7 and Figure 8 give the profiles of radial gas mean free path and number density. Through these two figures we can easily find that like the gas molecular mean free path including all direction’s collisions, the gas number density does not affect the radial gas molecular mean free path in the same way as it affects the gas molecular mean free path in the unbounded space.

### 2.5. The Body Force’s Effects on the Gas Molecular Mean Free Path

**Figure 9:**
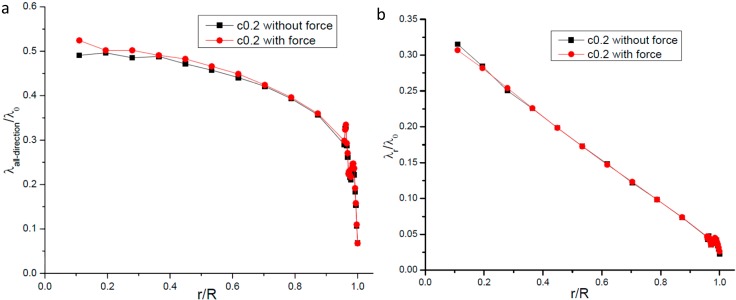
Comparison of simulation results whether the body force is added on every gas molecule. (**a**) gas molecular mean free path including all directions’ collisions; (**b**) radial gas molecular mean free path.

In our simulation runs one constant force was added on every molecule in the system in order to generate macroscopic gas flow. In this section we will study the effects by the body force on our simulation results. We take the nanopore whose radius is 16.06 nm and the value of *c* is 0.2 as an example to compare the difference between simulation results when the body force is added on or absent from every gas molecule. Figure 9 give our comparison results, from Figure 9 we can clearly see that the body force acted on every molecule in the simulation system has no obvious effects on the simulation results of both *λ_r_* and *λ_all−direction_*. Similar results are also obtained for other nanopore radii that we studied. This result is physically sound because the gas molecular mean free path is only dependent on the gas molecules’ relative motions and has nothing to do with molecular mass motion, which is generated by the body force.

## 3. Numerical Experimental Section

### 3.1. Simulation Model

**Figure 10:**
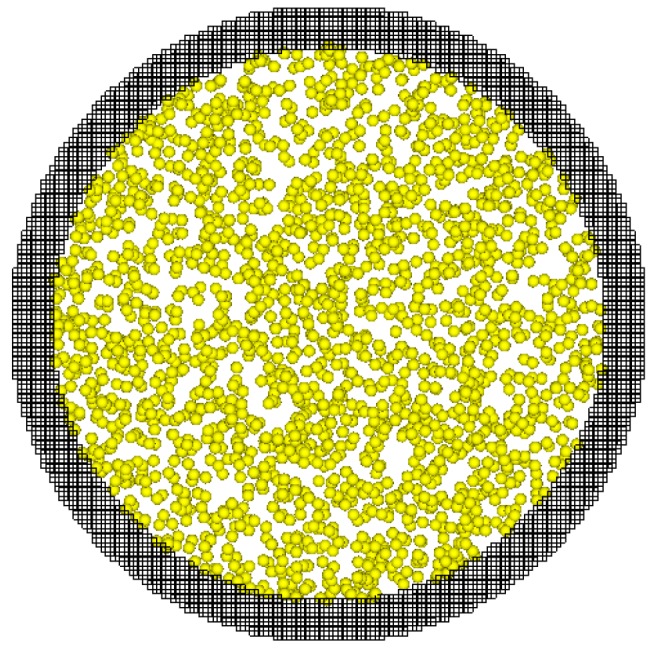
Cross-section snapshot of the particle locations in the nanopores of the MD (molecular dynamics) simulations.

In our present paper, molecular dynamics simulations are used to study the gas molecular free path in nanopores. The physical model for MD simulations is illustrated in Figure 10. The gas molecules are represented by the dots in Figure 10 and the nanopore is represented by the dark zone. In our simulation studies, argon was chosen as the representative gas because the Lennard-Jones (L-J) potential is well known for argon. As far as the solid nanopore is concerned, there are usually two type models, one is the stationary solid atoms and another alternative system is composed by mobile solid atoms. If the mobile solid system is utilized the computation load would be very large because the motions of wall atoms must be calculated. The main function of the wall surface is to surround the gas molecules through the interactions between gas molecules and wall atoms. There is little difference between these stationary solid atoms and the real situations, but this paper highlights the mean free path of gas molecules and the wall solid atoms mainly serve as one potential wall to surround the gas molecules. In order to reduce the computational load, the present study keeps the solid atoms stationary and for the reason of simplicity the nanopore material is also set as argon, but in the solid state. The solid nanopore is composed of argon atoms that are arranged in terms of FCC (face center cube) structure. The length of the nanopore is fixed at 53 nm. The radius of the nanopores is changeable in our simulations to study the size effects on the gas molecular mean free path.

The MD simulation is based on the integration of Newton’s second law by appropriate integral methods such as the Verlet method. In MD method, the force between two molecules is described by the potential function, for argon Equation (4) gives the function.

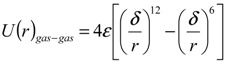
(4)
where *r* is the distance between two molecules, the energy parameter for argon is *ε* = 1.67 × 10^−21^ J and *δ* is the molecular length scale with value of 3.405 × 10^−10^ m [11].

The purpose of our studies on the gas molecular mean free path is to further understand the characteristics of gas flow at nanoscale, so the flow should exist. In this paper, the flow is generated by adding one constant body force on every molecule in the simulation domain. The value of body force is based on the following equation:

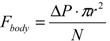
(5)
where *∆P* is the pressure difference between two ends of the nanopore, *r* is the radius of the nanopore and *N* is the total number of gas molecules in the nanopore.

The potential function between the gas and solid atoms also uses equation like Equation (4) that is:

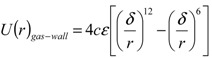
(6)
but with *ε* = 2.82 × 10^−21^ J and *δ* = 3.405 × 10^−10^ m where *c* is one variable parameter which represents the strength of interactions between gas molecules and wall atoms, when the value of *c* is larger the gas molecule-wall atom interaction strength is stronger. So for gas molecules without interaction with the wall, the force on every gas molecule is described as Equation (7) and for gas molecules interacting with the wall, is described as Equation (8).

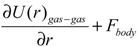
(7)

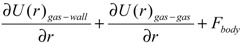
(8)

In our present studies, the initial positions of gas molecules are arranged according to FCC structure with the density of argon gas at the mean pressure of two ends of the nanopore at 300 K. The velocities of gas molecules were randomly initialized according to the Maxwell velocity distribution at 300 K and the total kinetic temperature is equal to the kinetic energy in the calculation domain:

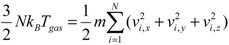
(9)

In Equation (9) *N* is the total number of gas molecules in the nanopore, which ranges from 2124 to 8592 when the diameter of the nanopore varies from 32.12 to 64 nm in this paper. *T_gas_* is always equal to 300 K and *k_B_* is the Boltzmann constant, *m* is the mass of gas molecule and *v*_*i*,*x*_, *v*_*i*,*y*_, *v*_*i*,*z*_ are gas molecule velocities in the *x*, *y* and *z* directions. Values for the initial force acted on each gas molecule were determined in terms of their initial positions *r_i_*(0).

### 3.2. Boundary Conditions

Due to the limitation of computational ability, it is impossible to simulate all of the molecules in the real flow system, so periodic boundary condition is applied to the flow direction, that is the *x* direction. In the radial direction of nanopore, no special boundary conditions are applied to gas molecules, because the movements of gas molecules are restricted by the wall of the nanopore.

### 3.3. Method of Gas Molecular Mean Free Path Calculation

According to the definition of mean free path, each molecule’s travel distance between two successive collisions is required to be recorded. But when the periodic boundary condition is applied, it is not appropriate to directly measure the travel distance of molecule as the difference between its current position and the position of its last collision. Instead, we calculate the molecule’s travel distance between two successive collisions as

*r = ∆t_c_ · v_current_* (10)

where *∆t_c_* is time difference between two successive collisions and *v_current_* is the current speed of the molecule.

The molecule’s travel distance between two successive collisions obtained through the above mentioned way is recorded to calculate the mean free path distribution *ψ*(*r*), which is the possibility a molecule has a free path between *r* and *r + dr*. Once *ψ*(*r*) is obtained, the effective gas molecular mean free path equals the value described as Equation (3).

In calculating the gas molecular mean free path, how to determine the collision is taking place is very important. The L-J potential is used in our studies, we judge the collision to take place if two molecules are closer together than a distance *r_col_*. In the present paper, the value of *r_col_* is set as *δ*. It should be noted that the molecule’s collisions with wall surface are also considered, we also judge the gas molecule-wall collision happens when the distance between the molecule and wall surface is less than *r_col_*. The validation of the *r_col_* is tested through the calculation of the mean free path of unconfined argon gas presented in Section 3.5.

### 3.4. Other Simulation Details

In our MD study, an equilibrium molecular dynamics simulation for gas with initially assigned number density at 300 K was conducted for the first 1,000,000 run steps and then the body force was added to every gas molecules in the nanopore. The temperature of gas needs to be controlled at 300 K, so the velocities of each gas molecules are controlled to maintain the desired temperature 300 K. In the present study, the Verlet method was used to solve the motion equation of all molecules in the simulation system and the atom list method was used to reduce simulation run time. The simulation run time step was set as 1.0 × 10^−15^ s.

### 3.5. Certification of the Methodology

To confirm the validity of our methodology for calculating the gas molecular mean free path using Equation (3), the unconfined argon gas molecular mean free path is calculated first. To simulate the unconfined gas, the cubic spatial geometry with periodic boundary conditions applied in three directions is adopted. To ensure the correctness of the simulation the side of the cubic is larger than the mean free path and two different cubic lengths are used to test the validity of our simulation results. These two different cubic lengths are 42.4 and 53.0 nm. The molecular density in the cubic is equal to the argon’s density at 0.2 MPa and 300 K.

**Figure 11:**
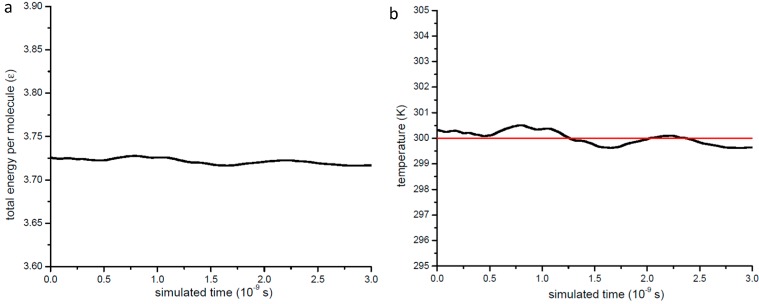
Thermal property’s variation with the simulation time for the case of unconfined gas at 0.2 MPa. (**a**) total energy per molecule; (**b**) temperature.

The MD simulations in the present paper are based on our own developed solver. To firstly test the validity of our MD solver, the total energy including potential energy and kinetic energy per molecule and temperature profile in the case of unconfined argon gas at 0.2 MPa is presented in Figure 11. From Figure 11 we can see that both the total energy per molecule and temperature fluctuate around one fixed value, but their deviations are very small compared with the desire value. According to the theory of statistical physics, any thermal property would show fluctuations which are related with the number of molecules in the system, so our simulation results are physically sound.

For the unconfined argon, our MD data relaxes to a mean free path value of 36.0 nm, which differs from the kinetic theoretical value only +0.55%. Our MD simulation also counts the number of multiple collisions and two body collisions, the results show that for unconfined argon gas at 0.2 MPa and 300 K the multiple collisions account for only 0.07% of total collisions, so the argon gas at 0.2 MPa can be regarded as ideal gas and the effects on the mean free path by the multiple collisions can be ignored. This illustrates that our method of calculating the mean free path through the integration of *ψ*(*r*) and the choice of the value *r_col_* are valid.

## 4. Conclusions

The gas molecular free path in naonpores with different radius and gas-wall interaction strengths was studied using molecular dynamics simulations and several conclusions have been obtained. The mean free path of all molecules in the nanopore is smaller than its counterpart value at macroscale space and is related with the nanopore’s radius and the gas-wall interaction strength. Smaller nanopore size and stronger gas-solid interactions would cause a shorter mean free path of all molecules. The radial gas molecular mean free path is smaller than the molecular mean free path including all direction’s collisions, which means the gas molecular motions in the nanopore are anisotropic. Both *λ_r_* and *λ_all−direction_* vary with the distance from the nanopore’s center. The lowest value of mean free path occurs at the wall surface of the nanopore. For the same nanopore, the relationship between *λ_r_* / *λ*_0_ and *r* / *R* can be described by the same linear function. Near the wall surface the gas-solid interaction plays a key role in determining both *λ_r_* and *λ_all−direction_*, the radius of the nanopore has only minor effects on them. For gas confined in nanopores, the gas number density does not affect the gas molecular mean free path in the same way as it does in the unbounded space.
